# GC-MS analysis of fatty acid metabolomics in RAW264.7 cell inflammatory model intervened by non-steroidal anti-inflammatory drugs and a preliminary study on the anti-inflammatory effects of NLRP3 signaling pathway

**DOI:** 10.1371/journal.pone.0290051

**Published:** 2023-08-15

**Authors:** Lin Chen, Luming Xie, Jing Zhang, Yifan Feng, Xia Wu

**Affiliations:** 1 Center for Drug Research and Development, Guangdong Pharmaceutical University, Guangzhou, Guangdong, China; 2 Guangdong Provincial Key Laboratory of Advanced Drug Delivery Systems and Guangdong Provincial Engineering Center of Topical Precise Drug Delivery System, Guangdong Pharmaceutical University, Guangzhou, Guangdong, China; Foshan University, CHINA

## Abstract

To explore the metabolomics of fatty acids and biological information of related markers in a RAW264.7 cell inflammation model. RAW264.7 macrophage inflammation model was induced by LPS, and RAW264.7 cells were treated with non-steroidal anti-inflammatory drugs (NSAIDs). The fatty acid compositions were identified by GC-MS, combined with standard product spectrum information and NIST (National Institute of Standards and Technology) database. Using chemometrics and Analysis of Variance (ANOVA), the components with VIP > 1 and P < 0.05 were selected as significant difference markers, and combined with biological methods to explore the biological significance of them. GC-MS identified 21 fatty acids in RAW264.7 cells, and screened significant difference biomarkers in each group. Among these biomarkers, C20:5 and C22:6 had significant changes in pairwise comparison among each group. Through ELISA, polymerase chain reaction (PCR) and Western Blot methods, the mRNA and protein expressions of IL-1β, NLRP3, GPR120 and β-Arrestin-2 were up-regulated after RAW264.7 cells induced by LPS and nigericin, and decreased after drug intervention. It indicated that the signal pathway centered on NLRP3 inflammasome was involved in the anti-inflammatory process of ibuprofen. It was the first time to study fatty acid metabolomics in RAW264.7 cell inflammatory model by GC-MS combined with chemometrics. The anti-inflammatory mechanism of ibuprofen was explained from NLRP3 inflammasome perspective without precedent, which enriched the research on the signal pathway of ibuprofen anti-inflammatory mechanism.

## Introduction

Inflammation is a protective response that occurs when the body is infected or the tissue is damaged. Clinically, inflammation is closely related to many other diseases, such as depression [1], diabetes [2], and asthma [3] etc. Fatty acids play an important role in the human body. In addition to provide energy, fatty acids are also involved in metabolic disorders of various diseases and play an important role in innate immunity, such as inflammation [3], diabetes [4], cancer [5] and other diseases. Therefore, the study of fatty acid metabolism may help to explain the molecular mechanism of disease pathogenesis. Polyunsaturated fatty acids play an important role in the regulation of inflammation. Among them, ω-3 fatty acids can be converted into some pro-catabolic mediators to inhibit inflammasomes, such as IL-1β and NF-κB. And ω-3 fatty acids can inhibit the conversion of ω-6 fatty acids to pro-inflammatory substances such as Prostaglandins E2 (PGE2). Investigation of the biological functions of the fatty acids associated with inflammation might enable further clarification of the importance of fatty acids molecules in inflammation.

The main analytical techniques in metabolomics are nuclear magnetic resonance (NMR) and mass spectrometry (MS) [6–9]. NMR has the advantages of simple pretreatment and no damage to the sample [10, 11]. The low sensitivity and resolution limit its wider use. The commonly used MS detection techniques are liquid chromatography-mass spectrometry (LC-MS) and gas chromatography-mass spectrometry (GC-MS). LC-MS has been widely used in the analysis of metabolites in biological systems because of its strong selectivity and high sensitivity [11, 12]. However, this technique is not suitable for the analysis of trace levels of fatty acids [13]. GC-MS has the advantages of high sensitivity and reproducibility, and also has the standard library (such as NIST, Fiehn, Wiley), which facilitates the identification of metabolites by GC-MS [14]. Therefore, GC-MS could provide more information on biological changes in an organism.

Some studies have shown that NLRP3 is involved in the pathogenesis of many inflammatory disorders [15, 16]. NLRP3 inflammasome have gradually become the central target for the study of inflammatory signaling pathways. In the resting state, NLRP3 is inactive. It is only activated by stress, which promotes the release of inflammatory factors such as IL-1β and promotes the inflammatory process.

In this study, GC-MS was analyzed the changes of fatty acids in RAW264.7 cells for the first time. Three NSAIDs (aspirin, ibuprofen and meloxicam) were used to interfere with the inflammatory model, and combined with chemometrics to explore the changes of fatty acid components in the process of inflammation generation, development and regression. Combined with biological methods, verify whether the anti-inflammatory effect of ibuprofen is through the signal pathway with NLRP3 inflammasome as the central target, which provides a new target for the treatment of the disease.

## Materials and methods

### Reagents

RAW264.7 cell was derived from the cell bank of the Chinese Academy of Sciences. Fetal bovine serum (FBS), Dulbecco’s modified eagle medium (DMEM) and 0.25% trypsin were from Thermo Fisher Scientific (Gibco, USA). Double antibody and phosphate buffer solution (PBS) were from Danaher Corporation (Hyclone, USA). Methanol (mass spectrum grade) was purchased from CNW Technologies GmbH (Germany). Fatty acid methyl ester was purchased from Merck (Sigma-Aldrich, Germany). Aspirin, meloxicam and ibuprofen were all from Guangdong Institute of Food and Drug Control (Guangzhou, China). TRNzol total RNA extraction reagents were purchased from Tiangen Biotechnology Co., Ltd.. PrimeScript™ RT reagent Kit with DNA Eraser and SYBR® Premix Ex Taq™ II (TliRNaseH Plus) were purchased from TaKaRa Biomedical Technology (Beijing) Co., Ltd., and PVDF membrane was purchased from Merck (Millipore, Germany).

### Cell culture

The cells were cultured in the complete medium prepared according to the ratio of DMEM: FBS: double antibody = 89:10:1, in a humidified atmosphere containing 5% CO_2_ at 37°C, with the medium changed every two days. The cells were passaged when the cell coverage reached more than 80% and were in good condition. The cell line was stored at −80°C until use.

### Cell grouping

Cells were seeded into dishes at the density of 2×10^6^/ml and cultured approximately for 12 h. Then the cells were randomly divided into the blank group (K), the inflammation group (Y) and drug groups (aspirin group, A; ibuprofen group, B; meloxicam group, M). Groups A, B, M were cultured with aspirin (0.72 mg/ml), ibuprofen (0.2 mg/ml) and meloxicam (0.03 mg/ml) at the optimal concentration respectively and the group Y and group B were added with the same volume of complete medium. One hour later, the inflammation group and drug groups were added with LPS to the final concentration of 10 μg/ml, and equal-volume PBS was added in group K. All of them were incubated for 24 h under the conditions mentioned above. The optimal concentration of the above drugs is based on the previous experimental experience in the laboratory [17, 18].

### Measurement of NO production

The standards were prepared according to the instructions. 50 μl of the standards and the supernatant of each group of cells were added to the 96-well plate, and then 100 μl of Griess reagent was added respectively. The absorbance was measured at 546 nm.

### Fatty acid extraction

The cells were washed with PBS twice and then the cell suspension was centrifuged at 600*g* for 10 min. Remove PBS and add 0.6 ml methanol (including 0.04% BHT)-chloroform (v/v = 2:1) solution and 0.1 M HCl. The solution was vortex mixed for 1 min and centrifuged at 16,000*g* for 8 min. The lower phase containing the fatty acid was isolated and dried under a stream of N_2_. Subsequently, the above samples were subjected to methyl esterification of fatty acids using the boron trifluoride method. Before GC-MS analysis, the above samples were derivatized, and then redissolved with n-hexane, and 20 μl internal standards were added for further analysis.

### GC-MS conditions

In this experiment, TSQ GC-MS (from Thermo Fisher Scientific) system was applied for analysis. A special chromatographic column for fatty acids TR-FAME (30 mm × 0.25 mm × 0.25 μm) was used. The carrier gas was constant current, 1 ml/min, and injection volume was 1 μl. A temperature program was adopted, the initial temperature was 65°C, held for 1 min, increased to 160°C at 5°C /min, held for 2 min, increased to 220°C at 2°C /min, increased to 230°C at 10°C /min, held for 2 min. The ionization mode was EI, and the ionization voltage was 70 eV. The ion source temperature was 230°C, the recording time was 55 min. The spectrum was obtained in the range of m/z 50–500.

### Data analysis for GC-MS

The data were imported into SIMCA-P14.0 software, and the orthogonal partial least square discriminant analysis (OPLS-DA) was established. Combined with the ANOVA, the components with VIP > 1 and P < 0.05 were selected as significant difference markers.

### Cell viability assay

The cultured RAW264.7 cells were diluted to 5 × 10^4^/ml, 100 μl were inoculated into 96-well plates, and cultured in the above conditions for 24 h. 100 μl of nigericin (inducer of NLRP3) of different concentrations was added to the cells and cultured for 48 h. The prepared complete medium containing 10% CCK-8 was added and cultured in the incubator. After 1h, the absorbance was determined by enzyme labeling instrument at wavelength 450 nm.

### IL-1β analysis

The secretion of IL-1β in the supernatant of each group was measured according to the relevant operation procedures of IL-1β ELISA kit.

### Real-time quantitative polymerase chain reaction

The total RNA of the cell samples of each treatment group was extracted by Trizol method and reverse transcribed into cDNA, which was used as a template for PCR amplification. Primers were designed by Primer Premier 5.0 software and synthesized by GENEWIZ. The primer sequence is shown in Table 1. The reaction conditions were as follows: initial denaturation at 95°C for 30 s, denaturation at 95°C for 5 s, annealing at 60°C for 30 s, and collecting the fluorescence signal after 40 cycles. With GAPDH as the internal reference, the relative expression of the target gene mRNA in the sample was calculated by 2^- ΔΔCT^ method.

**Table 1:** Primer sequences used in PCR.

Primer name	Primer Sequence 5’-3’
NLRP3-F	CCAGACACTCATGTTGCCTGTTC
NLRP3-R	GAGGCTCCGGTTGGTGCTTA
GPR120-F	AGCCACCCTGTGCCCTACCT
GPR120-R	GAGCCGTGATTGTGCCGTTG
β-Arrestin-2-F	GACCAGAGGACACAGGGAAG
β-Arrestin-2-R	TGATGAIAAGCCGCACAGAG
GAPDH-F	CGTGTTCCTACCCCCAATGT
GAPDH-R	TGTCATCATACTTGGCAGGTTTCT

### Western blot

Cell samples of each group were collected and centrifuged at 4°C at 2000 rpm for 5 min. The supernatant was discarded, the cell lysis buffer was added, and the protein was collected by centrifugation. According to the protein quantitative data, the corresponding total protein samples and 5-fold protein gel electrophoresis buffer solution were added, and treated at 95°C for 10 min to denature the protein. After preparing the separation gel and concentrated gel, the denatured protein was separated by SDS-PAGE and transferred to PVDF membrane. The PVDF membrane was sealed with 5% skimmed milk powder at room temperature for one hour, and the first antibody (NLRP3, GPR12) was added and incubated overnight at 4°C. Wash the film 3 times with 1 × TBST, 10 min for each time, add horseradish peroxidase labelled goat anti-mouse second antibody working solution (1: 5000) and incubate at room temperature, avoid light for one hour, wash the film, expose, and use image J software to analyze the gray value.

### Statistical analysis

All experiments were performed with triplicate samples and repeated at least three times. Statistical analysis was performed with GraphPad Prism software version 8.0. The data are presented as mean ± standard deviation (SD), and statistical comparisons between groups were performed using one-way analysis of variance (ANOVA) compared by Student’s t-test. *P*<0.05 were considered statistically significant.

## Results

### Release of LPS-induced NO

**Fig 1:**
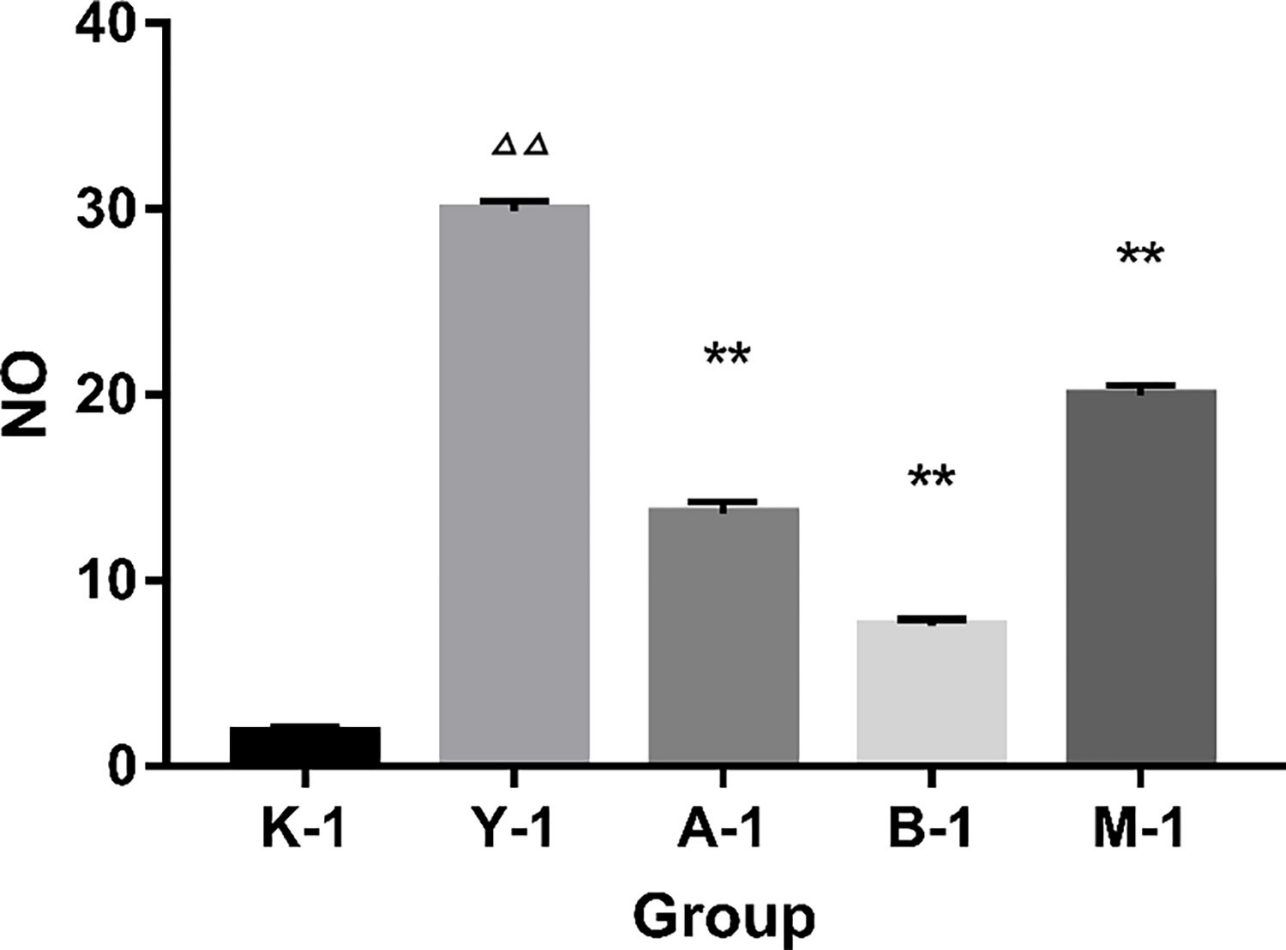
Amount of NO produced by RAW 264.7 murine macrophage cells. Note: K-1: normal group, Y-1: inflammation group, A-1 aspirin group, B-1ibuprofengroup. M-1: meloxicam group. Δ: compared with normal group (K), *: compared with model group (Y). Δ *P* < 0.05, ΔΔ *P* < 0.01, ΔΔΔ *P* < 0.001, * *P* < 0.05, ** *P* < 0.01, *** *P* < 0.001.

As shown in Fig 1, there was a significant difference in NO concentration between the inflammation group and the normal group, indicating that the inflammation model had been established successfully. There was also a significant difference in the concentration of NO between the drug administration groups and the inflammation group, suggesting that the drug intervention was successful.

### GC-MS method validation

At the beginning of the experiment, a Varian CP-WAX 52CB column was used, but not all fatty acids could be detected. Later, TR-FAME was used, which could not only separate the fatty acid components well, but also separate the cis/trans fatty acids. GC-MS has two ionization modes, EI (electron bombardment) and CI (chemical bombardment), CI can be selectively detected, but its reproducibility is poor, there is no standard library, while EI is non-selective, has a high ionization efficiency, good reproducibility, and there is a standard library. Therefore, EI ionization mode is usually used in GC-MS detection. For a combination of these reasons, we developed GC-MS conditions. The methodology of the method is verified from the aspects of specificity, precision, stability and repeatability. As shown in S1, S2 Figs, n-hexane was not retrieved in the target peak period, indicating that the n-hexane peak has been removed in the solvent delay time 5 min, and the internal standard does not affect the detection of the target substance, indicating that the specificity of the method is good. As shown in S1 to S5 Tables, the RSD values of precision, stability and repeatability are all less than 15%, which meets the requirements of biological detection.

### Identification of fatty acid

**Fig 2:**
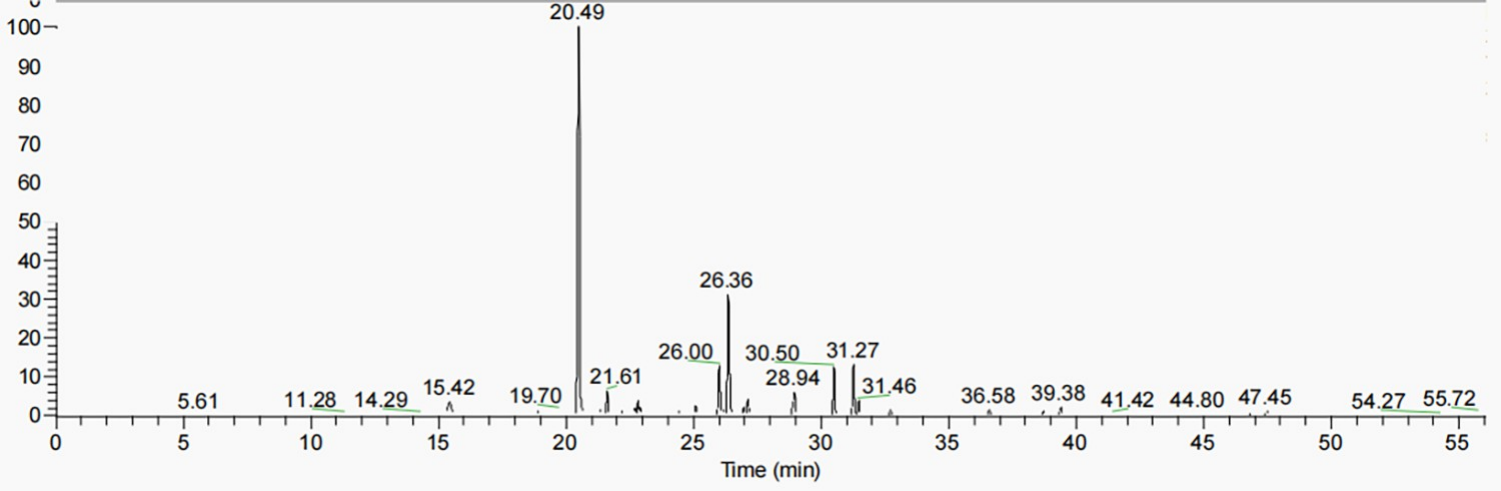
Fatty acid chromatogram of group B.

According to the fatty acid standard and NIST library, the fatty acid composition in RAW264.7 cells was identified (Table 2). The fatty acid chromatogram of group B is shown in Fig 2. In the process of identification, it was found that there was no significant difference in fatty acid composition among K, Y, A, B and M groups, but the content changed. As in the identification table, the fatty acid related rules can be found. The characteristic ions (m/z) of saturated fatty acids were 74, 87, 143, and 74 was the base peak. The characteristic ions (m/z) of monounsaturated fatty acids were 55, 69, 96, 110, and 55 was the base peak. The characteristic ions (m/z) of diunsaturated fatty acids were 67, 81, 95, and 67 is the base peak. The characteristic ions (m/z) of polyunsaturated fatty acids were 79, 91, 95, 105, and 79 was the base peak. The peak time of fatty acids was proportional to the length of carbon chain. The peak of fatty acids with short carbon chain was first, and the peak of fatty acids with long carbon chain was later. Trans-fatty acids peaks were earlier than cis-fatty acids.

**Table 2:** Fatty acid compositions in RAW264.7 cells.

NO.	t_R_ (min)	Name	Matching degree	Characteristic Fragment Ions
1	19.35	C12:0	917	74/87/214
2	22.83	C14:0	916	74/199/242
3	24.51	C15:0	911	74/213/256
4	26.35	C16:0	906	74/143/270
5	27.13	C16:1	924	55/194/268
6	28.33	C17:0	883	74/143/284
7	29.21	C17:1	920	55/250/282
8	30.5	C18:0	909	74/143/298
9	30.95	trans-C18:1	907	55/264/296
10	31.26	cis-C18:1	936	55/264/296
11	32.7	cis-C18:2	902	67/81/294
12	35.21	C20:0	883	74/283/326
13	36.06	C20:1	904	55/292/324
14	38.7	C20:3	858	79/150/320
15	39.37	C20:4	895	79/91/318
16	40.22	C22:0	898	74/143/354
17	41.14	C22:1	888	55/320/352
18	41.42	C20:5	877	79/105/316
19	45.26	C24:0	857	74/87/382
20	46.22	C24:1	887	55/97/348
21	47.45	C22:6	872	79/91/342

### Screening of potential biomarkers

S3 to S10 Figs show the OPLS-DA score chart and the VIP diagram between the pairwise comparisons of each group. The variables with VIP > 1 and ANOVA were taken as the biological results of fatty acid. Results showed that C22:6 (DHA) and C20:5 (EPA) were potential markers of fatty acid in inflammation group and ibuprofen groups (Table 3).

**Table 3:** Significant markers of fatty acids.

Grouping	Var ID (Primary)	VIP
K vs Y	trans-C18:1	1.41222
	C20:4	1.38603
	C16:1	1.38131
	C20:1	1.28362
	C24:0	1.22215
	C22:6	1.16907
	C 20:0	1.15156
	cis-Cl8:1	1.05464
	C20:3	1.0336
Y vs A	C24:0	1.59928
	C22:0	1.39913
	C17:l	1.33456
	C20:5	1.30302
	trans-C18:l	1.23759
	C16:l	1.1523
	C17:0	1.15741
	C20:0	1.07755
Y vs B	C24:0	1.47415
	C22:0	1.32177
	trans-C18:l	1.29983
	C20:3	1.24558
	C17:0	1.18387
	C15:0	1.15643
	cis-Cl8:1	1.11892
	C22:6	1.06566
	C20:5	1.03392
Y vs M	C22:0	1.43203
	C20:0	1.40208
	C24:0	1.3745
	C24:1	1.28363
	C20:5	1.1147
	C20:0	1.1036
	C12:0	1.04837

### Induced concentration and cell treatment of nigericin

NLRP3 inflammatory vesicle activation typically requires the involvement of two signals [19]. First signals include different TLR (toll-like receptor) ligands, such as LPS or other stimulatory signals, which upregulate the expression of proteins such as NLRP3 and Pro-IL-1, mainly through activation of the nuclear factor kB (NF-kB) pathway [20]. The second signal including nigericin, uric acid crystals, amyloid-β fibrils, and extracellular ATP [21]. Therefore, in this study, the combination of LPS and nigericin was chosen to induce the inflammation.

The experimental data were introduced into Graphpad Prism 7, and the cell survival rate was shown in S11 Fig, in which the abscissa represents the concentration of nigericin and the ordinate represents the cell survival rate. The results showed that the drug was more toxic to cells with increasing concentrations. The concentration of nigericin with a survival rate of more than 80% was selected as the induction concentration, which was 5 μM.

### Expression levels of IL-1β

**Fig 3:**
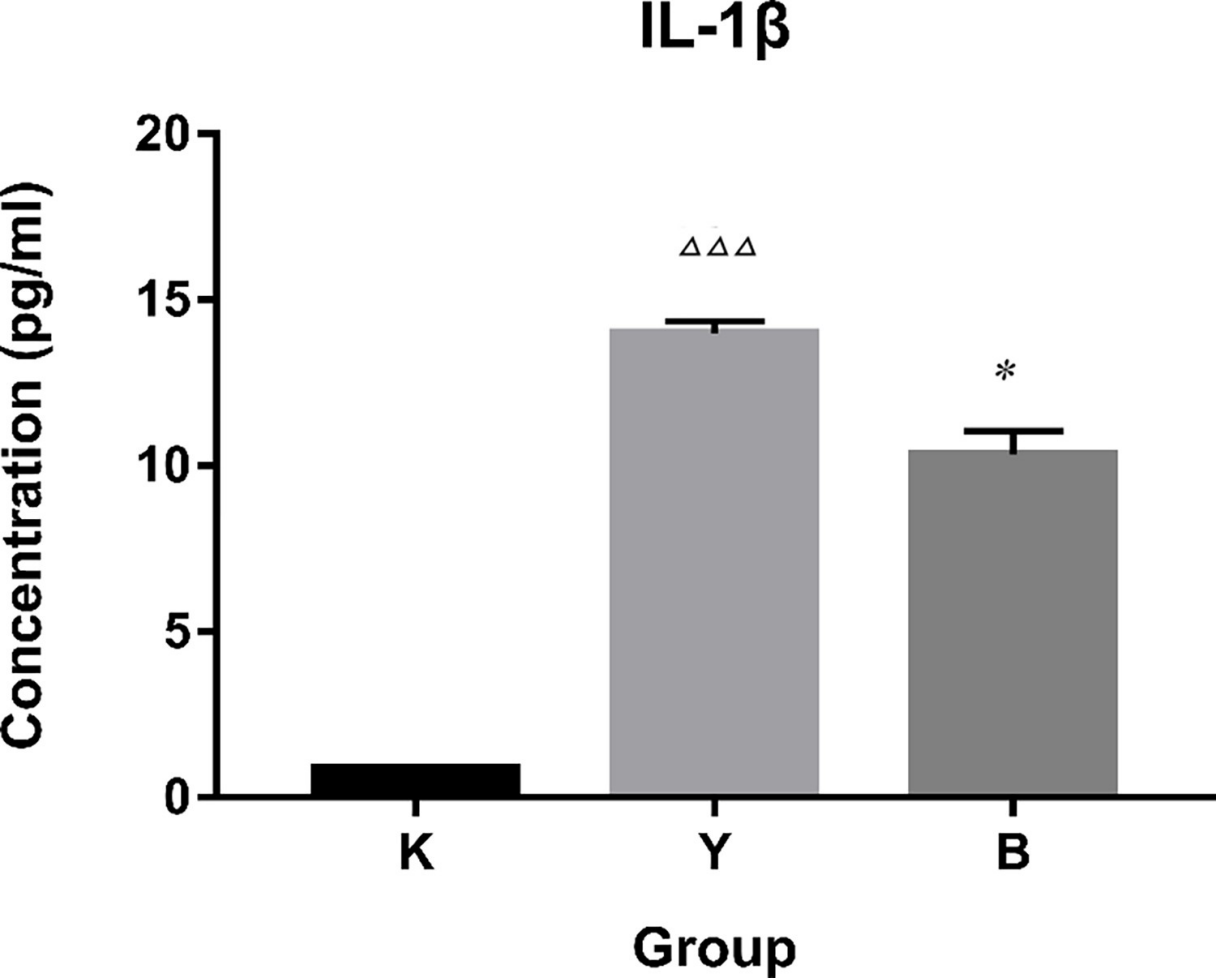
Expression levels of IL-1β assayed with ELISA. Note: K: normal group, Y: inflammation group, A: aspirin group, B: ibuprofen group. M: meloxicam group. Δ: compared with normal group (K), *: compared with model group (Y). Δ *P* < 0.05, ΔΔ *P* < 0.01, ΔΔΔ *P* < 0.001, * *P* < 0.05, ** *P* < 0.01, *** *P* < 0.001.

The results of ELISA showed that after stimulation of LPS and nigericin, the secretion of IL-1β in the supernatant of the inflammatory group was significantly higher than that in the blank group. The secretion of IL-1β in the supernatant of ibuprofen group was lower than that in the inflammatory group, indicating that ibuprofen could inhibit the secretion of IL-1β (Fig 3).

### mRNA expression of NLRP3, GPR120 and β-Arrestin-2

**Fig 4:**
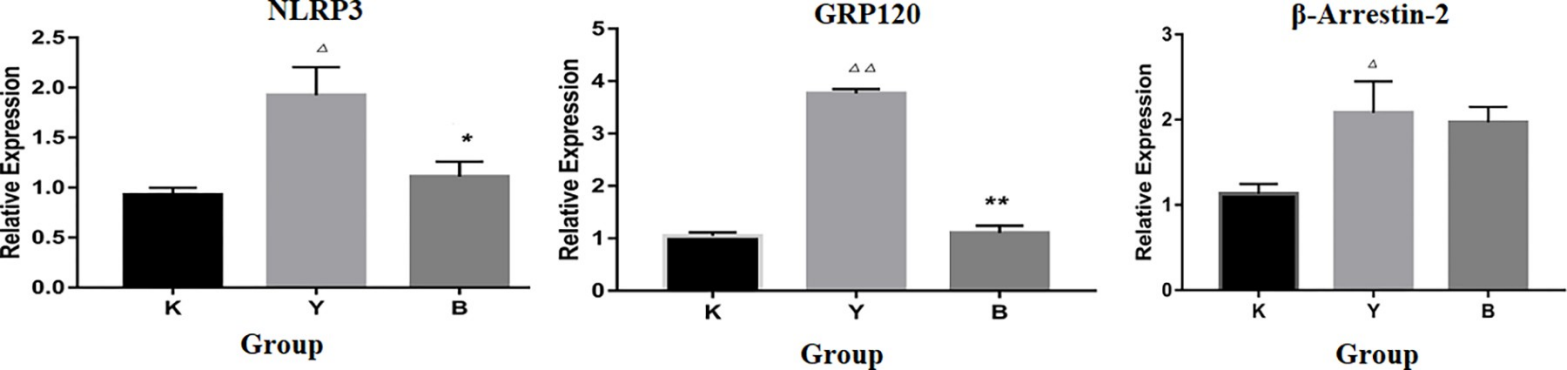
Effect of ibuprofen intervention on NLRP3, GPR120 and β-Arrestin-2 mRNA expression. **(**K: normal group, Y: inflammation group, A: aspirin group, B: ibuprofen group. M: meloxicam group. Δ: compared with normal group (K), *: compared with model group (Y). Δ *P* < 0.05, ΔΔ *P* < 0.01, ΔΔΔ *P* < 0.001, * *P* < 0.05, ** *P* < 0.01, *** *P* < 0.001).

The PCR results showed that NLRP3, GPR120 and their downstream genes β-Arrestin-2 in RAW264.7 cells increased after stimulation by LPS and nigericin, indicating that the NLRP3 signal pathway was activated and their expression decreased after the intervention of ibuprofen (Fig 4).

### Protein expression of NLRP3, GPR120 and β-Arrestin-2

**Fig 5:**
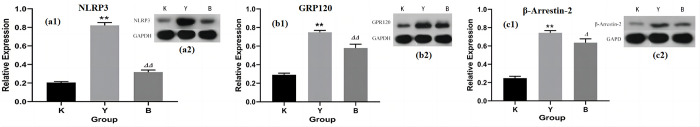
Effect of ibuprofen intervention on NLRP3, GPR120 and β-Arrestin-2 protein expression. **(a1)** Protein expression levels of NLRP3. **(a2)** Western Blot of NLRP3 expression. **(b1)** Protein expression levels of GPR120. **(b2)** Western Blot of GPR120 expression. **(c1)** Protein expression of β-Arrestin-2. **(c2)** Western Blot of β-Arrestin-2 expression. (K: normal group, Y: inflammation group, A: aspirin group, B: ibuprofen group. M: meloxicam group. Δ: compared with normal group (K), *: compared with model group (Y). Δ *P* < 0.05, ΔΔ *P* < 0.01, ΔΔΔ *P* < 0.001, * *P* < 0.05, ** *P* < 0.01, *** *P* < 0.001.).

NLRP3, GPR120 and β-Arrestin-2 in RAW264.7 cells increased after stimulation by LPS and nigericin, showing that the NLRP3 signal pathway was activated and their expression decreased after the intervention of ibuprofen (Fig 5). There were significant differences in protein expression between blank group and inflammatory group, inflammatory group and ibuprofen group, which were consistent with the trend of mRNA expression in PCR. It is suggested that ibuprofen has a potential therapeutic effect on the related inflammation caused by NLRP3 inflammatory bodies.

## Discussion

At present, many cellular and animal studies have shown that EPA and DHA can reduce inflammatory factors such as TNF and IL-1. It is now known that NF-κB is one of the main signal pathways involved in inflammation in the classical anti-inflammatory mechanism of fatty acids. When extracellular inflammatory stimulation occurs, IκB is phosphorylated, NF-κB cleavage, and then NF-κB dimer is activated and transferred into the nucleus, and the expression of inflammatory genes are up-regulated.

It is reported that GPR120 is involved in the inflammatory signaling pathway activated by long-chain fatty acids, while EPA and DHA inhibit the activation of NLRP3 inflammatory bodies by activating GPR120 [22–24]. When EPA and DHA inhibit the activation of NLRP3 inflammasome, β-arrestin-2 as the downstream protein of GPR120, can inhibit the activation of inflammatory factors by binding to NLRP3 [23, 25]. EPA and DHA were significant biomarkers of fatty acids in ibuprofen group and normal group, so it was speculated that NLRP3, GPR120 and β-arrestin-2, a signal pathway centered on NLRP3 inflammatory bodies, were involved in the anti-inflammatory effect of ibuprofen.

## Conclusion

In this study, 21 fatty acids in RAW264.7 cells were isolated and identified by GC-MS for the first time. The anti-inflammatory mechanism of ibuprofen was explained from NLRP3 inflammasome perspective without precedent, which enriched the research on the signal pathway of ibuprofen anti-inflammatory mechanism.

## Supporting information

S1 Fig Solvent specificity result diagram. (DOCX)

S2 Fig Specific result chart of internal standard. (DOCX)

S3 Fig Score chart of blank group and inflammatory group under OPLS-DA analysis. (DOCX)

S4 Fig Score chart of Inflammation and aspirin groups under OPLS-DA analysis. (DOCX)

S5 Fig Score chart of inflammation and ibuprofen groups under OPLS-DA analysis. (DOCX)

S6 Fig Score chart of inflammation and meloxicam groups under OPLS-DA analysis. (DOCX)

S7 Fig VIP diagram of blank group and inflammatory group under OPLS-DA analysis. (DOCX)

S8 Fig VIP diagram of inflammatory group and aspirin group under OPLS-DA analysis. (DOCX)

S9 Fig VIP diagram of inflammatory group and ibuprofen group under OPLS-DA analysis. (DOCX)

S10 Fig VIP diagram of inflammatory group and meloxicam group under OPLS-DA analysis. (DOCX)

S11 Fig Nigericin CCK-8 results. (DOCX)

S1 Table Results of intra-day precision inspection. (DOCX)

S2 Table Results of daytime precision investigation. (DOCX)

S3 Table Results of sample stability investigation. (DOCX)

S4 Table Results of investigation on the stability of the instrument. (DOCX)

S5 Table Results of repetitive investigation. (DOCX)
