# Supplementary figures and images for: GC-MS analysis of fatty acid metabolomics in RAW264.7 cell inflammatory model intervened by non-steroidal anti-inflammatory drugs and a preliminary study on the anti-inflammatory effects of NLRP3 signaling pathway
Source: PLoS One. 2023 Aug 15;18(8):e0290051. doi: 10.1371/journal.pone.0290051 (PMC10426916; doi:10.1371/journal.pone.0290051)

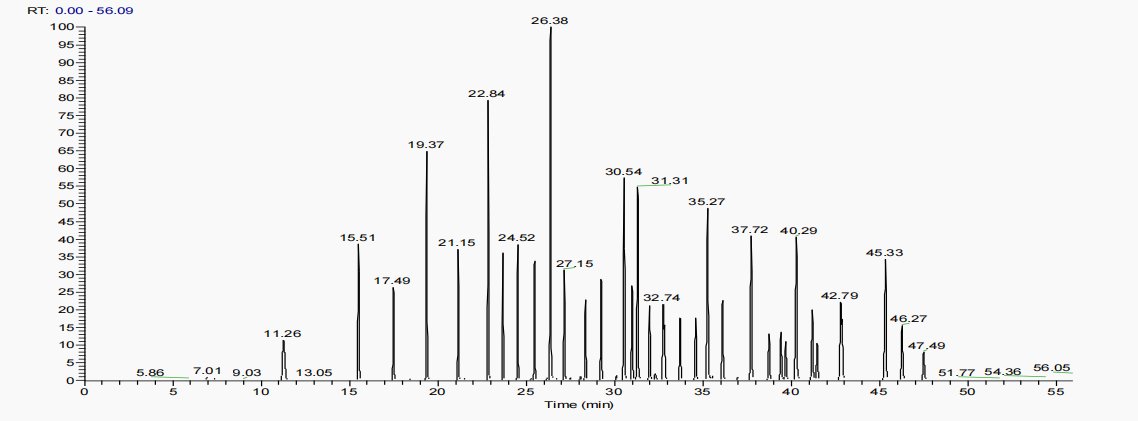


**Figure S1:** Solvent specificity result diagram

Supplement: S1 Fig — (DOCX) [file pone.0290051.s001.docx]

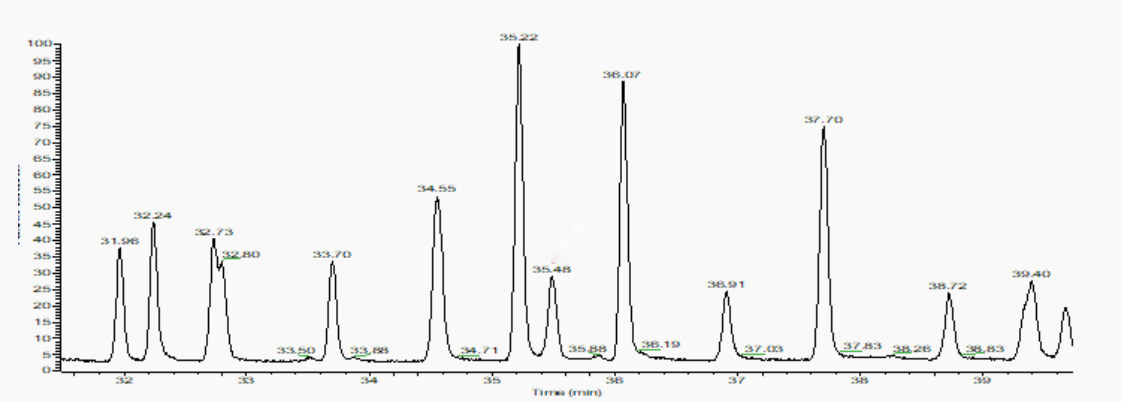


**Figure S2:** Specific result chart of internal standard

Supplement: S2 Fig — (DOCX) [file pone.0290051.s002.docx]

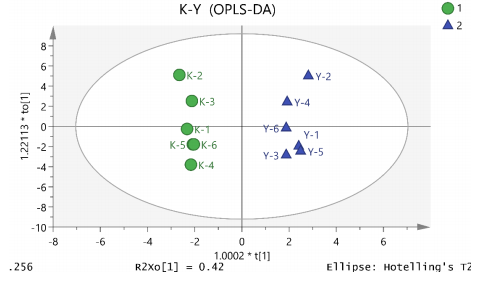


**Figure S3:** Score chart of blank group and inflammatory group under OPLS-DA analysis

Supplement: S3 Fig — (DOCX) [file pone.0290051.s003.docx]

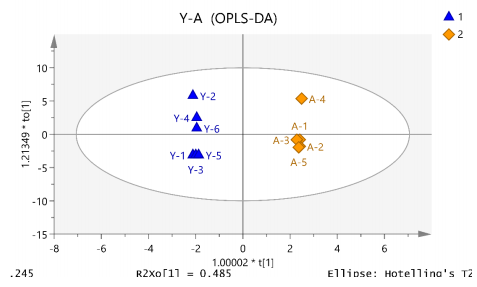


**Figure S4:** Score chart of Inflammation and aspirin groups under OPLS-DA analysis

Supplement: S4 Fig — (DOCX) [file pone.0290051.s004.docx]

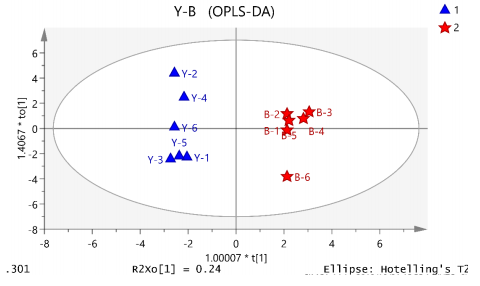


**Figure S5:** Score chart of Inflammation and ibuprofen groups under OPLS-DA analysis

Supplement: S5 Fig — (DOCX) [file pone.0290051.s005.docx]

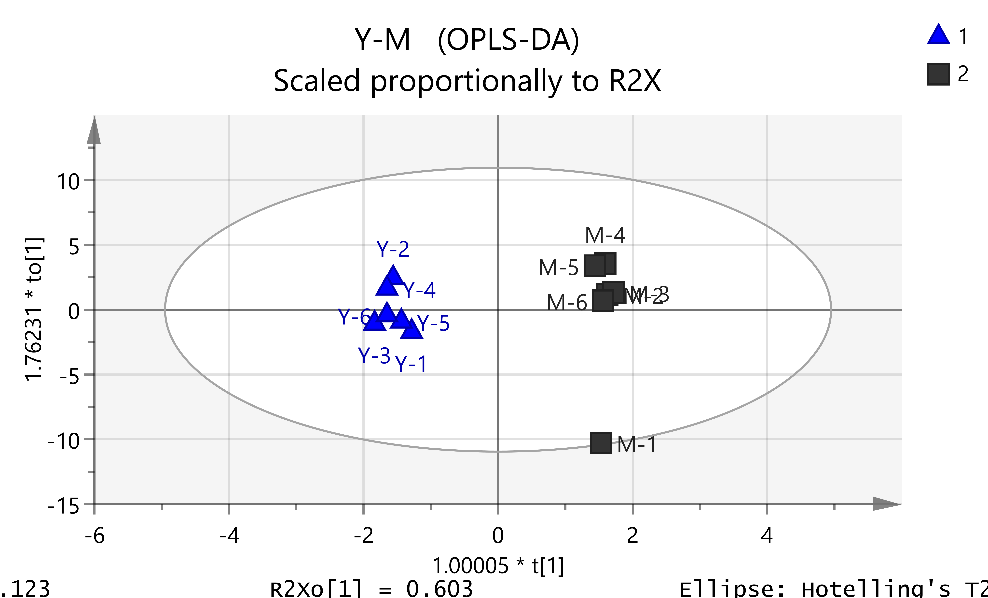


**Figure S6:** Score chart of Inflammation and meloxicam groups under OPLS-DA analysis

Supplement: S6 Fig — (DOCX) [file pone.0290051.s006.docx]

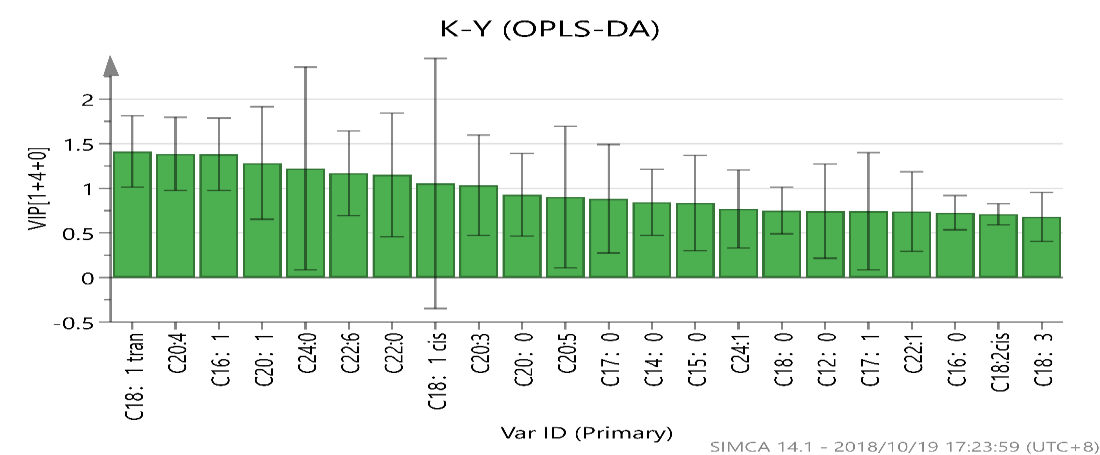


**Figure S7:** VIP diagram of blank group and inflammatory group under OPLS-DA analysis

Supplement: S7 Fig — (DOCX) [file pone.0290051.s007.docx]

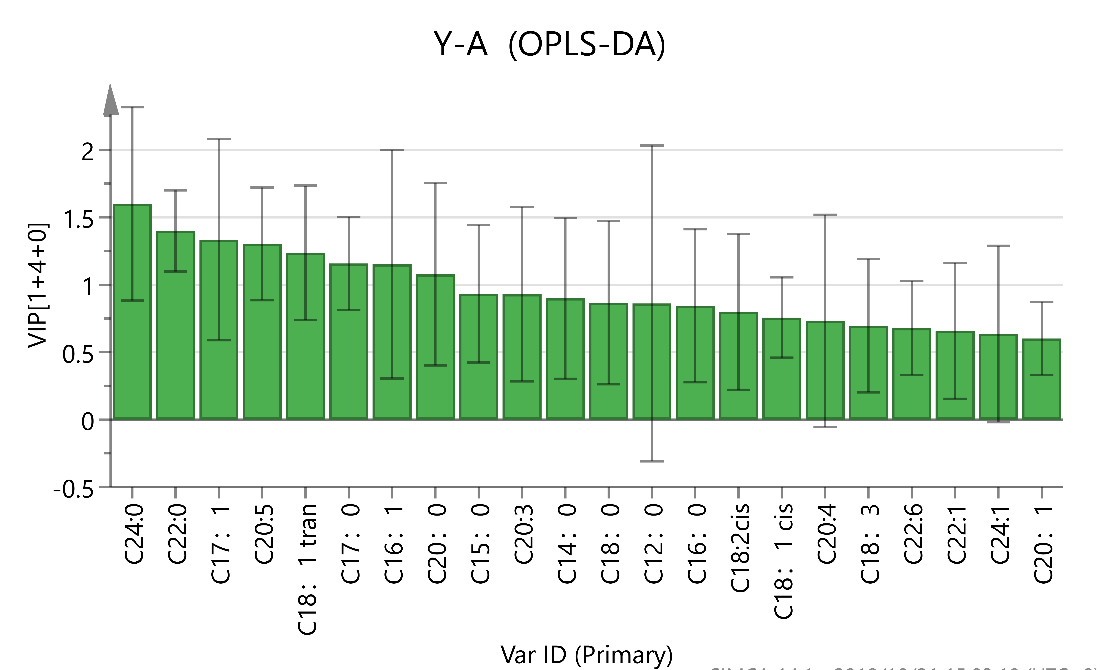
 **Figure S8:** VIP diagram of inflammatory group and aspirin group under OPLS-DA analysis

Supplement: S8 Fig — (DOCX) [file pone.0290051.s008.docx]

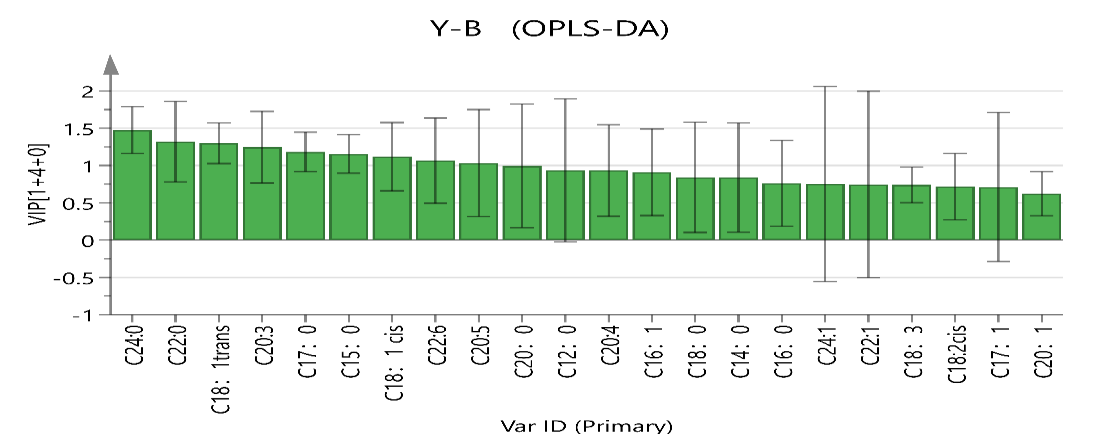
 **Figure S9:** VIP diagram of inflammatory group and ibuprofen group under OPLS-DA analysis

Supplement: S9 Fig — (DOCX) [file pone.0290051.s009.docx]

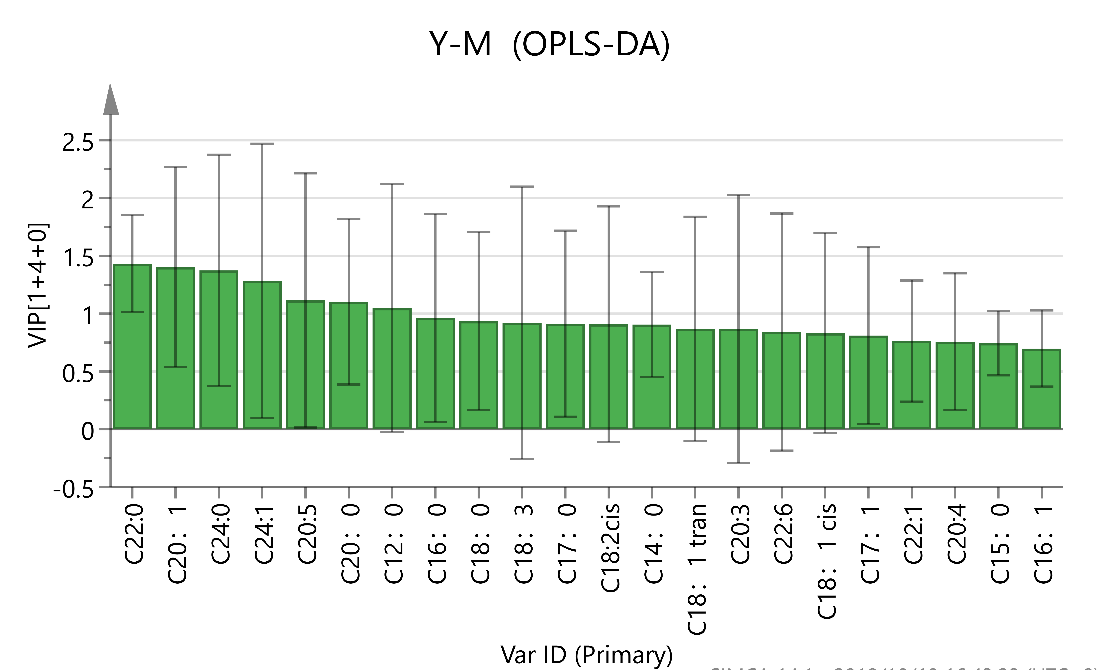
 **Figure S10:** VIP diagram of inflammatory group and meloxicam group under OPLS-DA analysis

Supplement: S10 Fig — (DOCX) [file pone.0290051.s010.docx]

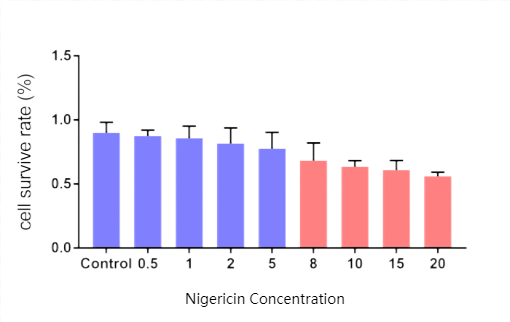


**Figure S11:** Nigericin CCK-8 Results

Supplement: S11 Fig — (DOCX) [file pone.0290051.s011.docx]
