# Supplementary material for: GC-MS analysis of fatty acid metabolomics in RAW264.7 cell inflammatory model intervened by non-steroidal anti-inflammatory drugs and a preliminary study on the anti-inflammatory effects of NLRP3 signaling pathway
Source: PLoS One. 2023 Aug 15;18(8):e0290051. doi: 10.1371/journal.pone.0290051 (PMC10426916; doi:10.1371/journal.pone.0290051)
Supplement: S1 Table — (DOCX) [file pone.0290051.s012.docx]

**Table S1** Results of intra-day precision inspection

| Name | 1 | 2 | 3 | 4 | 5 | 6 | RSD |
| --- | --- | --- | --- | --- | --- | --- | --- |
| C15:0 | 0.3734 | 0.3736 | 0.3629 | 0.3482 | 0.3573 | 0.3609 | 0.0269 |
| C16:1 | 0.9340 | 0.9240 | 0.8828 | 0.9202 | 0.9080 | 0.9904 | 0.0387 |
| C18:1trans | 5.4438 | 5.6103 | 5.2689 | 5.4524 | 5.2131 | 5.4710 | 0.0268 |
| C18:2cis | 0.5205 | 0.5781 | 0.5253 | 0.5234 | 0.5144 | 0.5275 | 0.0437 |
| C22:1 | 0.1231 | 0.1358 | 0.1297 | 0.1365 | 0.1443 | 0.1454 | 0.0628 |
| C24:0 | 0.2291 | 0.2504 | 0.2523 | 0.2495 | 0.2531 | 0.2886 | 0.0759 |
