# Supplementary material for: GC-MS analysis of fatty acid metabolomics in RAW264.7 cell inflammatory model intervened by non-steroidal anti-inflammatory drugs and a preliminary study on the anti-inflammatory effects of NLRP3 signaling pathway
Source: PLoS One. 2023 Aug 15;18(8):e0290051. doi: 10.1371/journal.pone.0290051 (PMC10426916; doi:10.1371/journal.pone.0290051)
Supplement: S2 Table — (DOCX) [file pone.0290051.s013.docx]

**Table S2** Results of daytime precision investigation

| Name | The first day | | The scend day | | The third day | | RSD | |
| --- | --- | --- | --- | --- | --- | --- | --- | --- |
|  | t_R_(min) | S | t_R_(min) | S | t_R_(min) | S | t_R_(min) | S |
| C16:1 | 27.13 | 0.7850 | 27.13 | 0.8828 | 27.14 | 0.8770 | 0.0002 | 0.0647 |
| C18:0 | 30.53 | 12.8717 | 30.53 | 13.5105 | 30.53 | 12.0558 | 0.0000 | 0.0569 |
| C22:1 | 41.14 | 0.1280 | 41.14 | 0.1295 | 41.15 | 0.1403 | 0.0001 | 0.0505 |
| C20:5 | 41.42 | 0.1502 | 41.43 | 0.1666 | 41.44 | 0.1695 | 0.0002 | 0.0643 |
| C24:0 | 45.25 | 0.2571 | 45.23 | 0.2955 | 45.26 | 0.3038 | 0.0003 | 0.0872 |
| C24:1 | 46.20 | 0.1002 | 46.22 | 0.1146 | 45.21 | 0.1068 | 0.0002 | 0.0671 |
