# Supplementary material for: GC-MS analysis of fatty acid metabolomics in RAW264.7 cell inflammatory model intervened by non-steroidal anti-inflammatory drugs and a preliminary study on the anti-inflammatory effects of NLRP3 signaling pathway
Source: PLoS One. 2023 Aug 15;18(8):e0290051. doi: 10.1371/journal.pone.0290051 (PMC10426916; doi:10.1371/journal.pone.0290051)
Supplement: S3 Table — (DOCX) [file pone.0290051.s014.docx]

**Table S3** Results of sample stability investigation

| Name | 0 h | 2 h | 4 h | 8 h | 12 h | 48 h | RSD |
| --- | --- | --- | --- | --- | --- | --- | --- |
| C12:0 | 0.0468 | 0.0459 | 0.0410 | 0.0437 | 0.0417 | 0.0400 | 0.0639 |
| C20:1 | 0.1413 | 0.1473 | 0.1344 | 0.1344 | 0.1392 | 0.1454 | 0.0334 |
| C20:3 | 0.2399 | 0.2424 | 0.2323 | 0.2575 | 0.2572 | 0.2408 | 0.0414 |
| C20:4 | 0.9383 | 0.9535 | 0.9637 | 1.0257 | 0.9699 | 1.0042 | 0.0336 |
| C22:1 | 0.1521 | 0.1431 | 0.1295 | 0.1608 | 0.1525 | 0.1559 | 0.0750 |
| C22:6 | 0.5262 | 0.5598 | 0.5728 | 0.5813 | 0.5750 | 0.6061 | 0.0463 |
