# Supplementary material for: GC-MS analysis of fatty acid metabolomics in RAW264.7 cell inflammatory model intervened by non-steroidal anti-inflammatory drugs and a preliminary study on the anti-inflammatory effects of NLRP3 signaling pathway
Source: PLoS One. 2023 Aug 15;18(8):e0290051. doi: 10.1371/journal.pone.0290051 (PMC10426916; doi:10.1371/journal.pone.0290051)
Supplement: S4 Table — (DOCX) [file pone.0290051.s015.docx]

**Table S4** Results of investigation on the stability of the instrument

| Name | 1 | 2 | 3 | 4 | RSD |
| --- | --- | --- | --- | --- | --- |
| C11:0 | 0.5878 | 0.5539 | 0.5378 | 0.5341 | 0.0442 |
| C14:1 | 1.3534 | 1.3218 | 1.3145 | 1.3013 | 0.0167 |
| C15:0 | 0.6246 | 0.6376 | 0.6281 | 0.6298 | 0.0087 |
| C16:0 | 2.0249 | 2.0718 | 2.0207 | 2.0446 | 0.0114 |
| C18:0 | 1.1491 | 1.2400 | 1.2407 | 1.2223 | 0.0358 |
| C20:0 | 0.9805 | 1.0775 | 1.1066 | 1.0839 | 0.0526 |
