# Supplementary material for: GC-MS analysis of fatty acid metabolomics in RAW264.7 cell inflammatory model intervened by non-steroidal anti-inflammatory drugs and a preliminary study on the anti-inflammatory effects of NLRP3 signaling pathway
Source: PLoS One. 2023 Aug 15;18(8):e0290051. doi: 10.1371/journal.pone.0290051 (PMC10426916; doi:10.1371/journal.pone.0290051)
Supplement: S5 Table — (DOCX) [file pone.0290051.s016.docx]

**Table S5** Results of repetitive investigation

| Name | 1 | 2 | 3 | 4 | 5 | 6 | RSD |
| --- | --- | --- | --- | --- | --- | --- | --- |
| C16:1 | 0.8722 | 0.8802 | 0.9437 | 0.7048 | 0.8770 | 0.8870 | 0.0939 |
| C18:1 | 5.0837 | 6.2870 | 5.3727 | 4.4362 | 5.1323 | 4.7083 | 0.1238 |
| C20:3 | 0.2240 | 0.2279 | 0.2460 | 0.1704 | 0.2589 | 0.2217 | 0.1348 |
| C20:4 | 0.8763 | 0.9020 | 0.9404 | 0.7537 | 0.9547 | 0.8649 | 0.0816 |
| C20:5 | 0.1379 | 0.1828 | 0.1574 | 0.1417 | 0.1695 | 0.1636 | 0.1070 |
| C22:6 | 0.4914 | 0.5504 | 0.5241 | 0.4370 | 0.5654 | 0.5312 | 0.0898 |
